# Childhood glycaemic control has an enduring effect on the lifetime risk of microvascular complications in type 1 diabetes mellitus

**DOI:** 10.1186/1687-9856-2015-S1-O37

**Published:** 2015-04-28

**Authors:** Mary White, Matt Sabin, Costan Magnusson, Michele O’Connell, Fergus Cameron

**Affiliations:** 1 Department of Endocrinology & Diabetes and Centre for Hormone Research, Murdoch Childrens Research Institute at The Royal Children’s Hospital, Melbourne, VIC, Australia; 2 Menzies Research Institute Tasmania, University of Tasmania, Hobart, TAS, Australia

## 

The development of diabetes-related microvascular complications in type 1 diabetes (T1DM) is known to be related to glycaemic control, but the degree to which variations in HbA1c across the lifetime contributes to this risk is unknown. Our hypothesis was that individuals with poor control in childhood and subsequent improved control in adulthood would still have an increased risk of severe diabetes-related complications when compared with individuals who achieved good control throughout the lifecourse. This study aimed to investigate this premise in a cohort for whom serial lifetime glycaemic data are available. The study population comprised children diagnosed with T1DM <18 years at The Royal Children’s Hospital (Melbourne) who transitioned to the Royal Melbourne Hospital for ongoing (adulthood) care. Data were collected through BioGrid Australia, a biorepository allowing inter-institutional data linkage, and included demographics as well as serial HbA1c and complication data. ‘Severe complications’ were defined as severe diabetes-related eye disease, renal failure, ulceration/amputation or death. Glycaemic control was categorised according to HbA_1c_ changes over time, based on median values throughout the lifecourse; “Optimal” (≤8.2% throughout lifecourse), “Improving” (>8.2% in childhood, ≤8.2% in adulthood), “Worsening” (≤8.2% in childhood, >8.2% in adulthood), “Poor” (>8.2% throughout lifecourse). A total of 503 (male=253) individuals were identified, diagnosed 1975-2010. At the time of follow up, mean (SD) age was 27.9 (6.2) years and median (IQR) duration of diabetes was 17.8 (12.2, 23.2) years. Severe complications were documented in 26 (5.2%) and were associated with mean HbA_1c_ at age 16-30 years (<0.05) and intra-individual lifetime glycaemic variability expressed as HbA_1c_SD (p=0.02). The relative risk (95% confidence interval) of developing severe complications in the improving, worsening and poor groups was 14.9 (1.7-130.9, p=0.01, n=50), 12.5 (1.4-109.4, p <0.01, n=60) and 15.4 (2.1-114.8, p <0.01, n=206) respectively when compared to the optimal group (n=187). In conclusion, the overall rate of severe complications is low in this cohort despite the lifecourse poor glycaemic control demonstrated in 40.1%. Our findings demonstrate that poor glycaemic control in childhood has a lasting effect on the development of severe microvascular complications in adulthood.

